# Isolated Pancreatic Metastasis From Renal Clear Cell Renal Cell Carcinoma 29 Years After Radical Nephrectomy

**DOI:** 10.7759/cureus.54973

**Published:** 2024-02-26

**Authors:** Sho Fujiwara, Nozomi Koyamada, Ryuichi Nishimura, Koji Miyazawa, Shukichi Miyazaki

**Affiliations:** 1 Department of Surgery, Iwate Prefectural Chubu Hospital, Kitakami, JPN

**Keywords:** hepatic-bilio-pancreatic surgery, renal cancer, renal cancer, pancreatico-duodenectomy (pd), renal cell cancer metastasis, metastatic pancreas cancer

## Abstract

Isolated metastatic tumors of the pancreas from other origins are only 2-3% of pancreatic cancers, and renal cell carcinoma is the most common origin of metastasis. It is challenging to differentiate between pancreatic tumors and those with a history of renal cancer to optimize treatment and management of this tumor. Here, we present a case of isolated renal cell cancer metastasis to the pancreas, which occurred 29 years after the radical nephrectomy. Surgical resection and pancreatectomy is a feasible treatment because of the low rate of complication and favorable prognosis. However, isolated metastatic pancreatic cancer from renal cell cancer is rare and has relatively high risk of recurrence. Therefore, a larger sample size is necessary to evaluate long-term oncologic outcomes and to optimize diagnostic and therapeutic strategies.

## Introduction

Metastatic tumors of the pancreas from other origins on the pancreas are rare, accounting for only 2-3% of pancreatic cancers [1,2]. Renal cell carcinoma is the most common origin of metastasis [1]. Approximately 9.7% of metastatic renal cell carcinoma cases involve metastasis to the pancreas [3]. Therefore, it is crucial to differentiate between pancreatic tumors and those with a history of renal cancer to optimize treatment of metastatic renal cancer to the pancreas. The therapeutic strategy is variable and multidisciplinary based on the tumor progression, such as surgical resection, chemotherapy, and immune checkpoint inhibitors [3]. Recent advancements in immune checkpoint inhibitors have significantly improved the prognosis of renal cell carcinoma. However, preoperative diagnosis of pancreatic metastases from renal cell cancer is sometimes challenging due to the similarity of imaging findings. Here, we present a case of isolated renal cell cancer metastasis to the pancreas, which occurred 29 years after the radical nephrectomy.

## Case presentation

A 62-year-old man with a history of hypertension and right radical nephrectomy 29 years ago for clear cell renal cell carcinoma was admitted to our hospital with anemia, hematemesis, and melena. Laboratory data showed mild anemia, but tumor markers, including carcinoembryonic antigen and carbohydrate antigen 19-9, were unremarkable.

**Figure 1:**
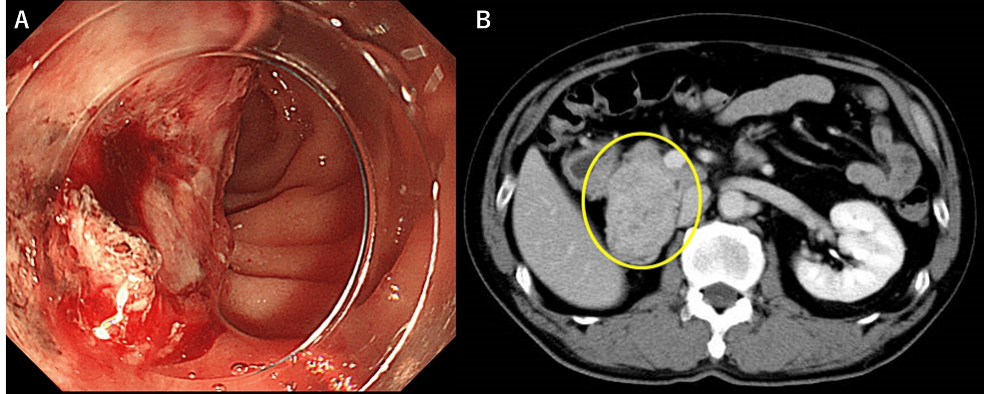
Endoscopic image of duodenum and contrast-enhanced abdominal computed tomography (A) Endoscopic image of duodenum. Invasion from a tumor of the pancreatic head, (B) Computed tomography showing hypervascular tumor of pancreas head.

An upper gastrointestinal endoscopy revealed a hemorrhagic invasive lesion in the duodenum. The biopsy was inconclusive due to necrotic tissue, but invasion from the pancreatic head tumor was consistent (Figure 1A). Contrast-enhanced computed tomography revealed a 50 mm enhanced tumor of the pancreatic head with 7 mm lymphadenopathy (Figure 1B).

**Figure 2:**
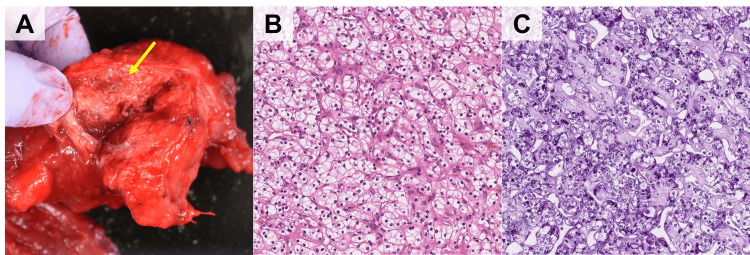
Gross and pathological findings of resected specimens (A) Gross presentation of surgical specimen. Yellow arrow shows the tumor of pancreas head, (B) H&E staining, magnification x200. Histopathological finding of the tumor showing clear cytoplasm with small thin walls, (C) Periodic acid–Schiff staining, magnification x200. Periodic acid–Schiff staining demonstrated cytoplasm containing lipid and glycogen.

The differential diagnoses based on the CT scan included neuroendocrine and metastatic tumors from renal cancer. Pancreaticoduodenectomy, Whipple procedure, with lymphadenectomy was performed because there were no other metastatic sites (Figure 2A). We were not able to diagnose. Histopathological finding of the tumor was clear cytoplasm with small thin walls, and periodic acid-Schiff staining demonstrated cytoplasm containing lipid and glycogen (Figure 2B-2C). These pathological findings were consistent with a metastatic pancreatic tumor from renal clear cell carcinoma. The patient was discharged without complications. We are following up with CT scan every year and has remained recurrence-free for seven years since the surgery.

## Discussion

We reported a case of isolated metastatic tumor of the pancreas from renal cell cancer 29 years post-nephrectomy. Metastatic pancreatic cancer is an extremely rare occurrence [1,2]. Our findings highlight two important clinical issues: the importance of distinguishing pancreatic tumors from those with a history of renal cancer, and the need to optimize treatment and management of metastatic renal cancer to the pancreas.

Computed tomography is a useful tool for diagnosing metastatic pancreatic cancer from renal cell cancer, even in asymptomatic cases [4]. In our case, we were not able to diagnose precisely by CT scan and the differential diagnosis was neuroendocrine tumor or metastatic renal cancer, because he underwent radical nephrectomy more than 20 years ago. Contrast-enhanced computed tomography can detect renal cell carcinoma metastases as an enhanced mass, similar to primary renal cancer, and the enhancement can persist into the late phase [5]. Pancreatic lymph node metastasis is found in 20-30% of cases, but metastatic pancreatic cancer from renal cancer has a favorable prognosis after radical resection [6]. In our case, the bleeding was caused by the invasion of tumor. The patient underwent nephrectomy 29 years ago, and the clinical stage at that time is unknown. A previous report suggested that metachronous metastasis occurred in 91.6% of cases, and T1 renal cancer accounted for 9.8% [4]. Moreover, median relapse interval of renal cell carcinoma is reported to be 15 to 18 months [6]. It is important to consider the possibility of metachronous metastasis from renal cancer, even if the TNM category indicates a favorable staging.

Surgical resection is still feasible as a treatment option for isolated pancreatic metastasis of renal cell carcinoma, although previous report suggests the effect of tyrosine kinase inhibitors to improve the overall survivals of renal cell carcinoma metastasized to pancreas [7-10]. Currently, we have multiple treatment options such as surgery, vascular endothelial growth factor receptor-targeted therapy, and immunotherapy [11]. Even if patients have extrapancreatic metastases, their five-year overall survival is approximately 50% after the surgical resection [8]. Furthermore, the morbidity and mortality rates for the operation of metastatic pancreatic cancer are relatively low [12]. Therefore, radical resection, including lymphadenectomy, is feasible and contributes to a better prognosis [9,10]. As a result, we decided to perform a pancreaticoduodenectomy with lymphadenectomy. However, it is important to thoroughly follow up with these patients, as the adjusted relative risk of repeated recurrence in patients who have undergone extended pancreatectomy is significantly higher than synchronous metastasis of pancreas [13].

## Conclusions

Isolated metastatic tumor of the pancreas from renal cell cancer is rare but renal cancer is the most common origin of metastatic pancreatic cancer. Pancreatectomy is a feasible treatment option due to low complication rates and favorable prognosis, despite the relatively high risk of recurrence. However, the treatment strategies need optimization to reduce the risk of recurrence and advance of systemic therapy.
